# Editorial: Integrative Physiology: Systemic Hypertension and Respiratory-Sympathetic Coupling

**DOI:** 10.3389/fphys.2022.841001

**Published:** 2022-01-26

**Authors:** Thiago S. Moreira, Ana C. Takakura, Eduardo Colombari

**Affiliations:** ^1^Department of Physiology and Biophysics, Instituto de Ciencias Biomedicas, Universidade de São Paulo (USP), São Paulo, Brazil; ^2^Department of Pharmacology, Instituto de Ciencias Biomedicas, Universidade de São Paulo (USP), São Paulo, Brazil; ^3^Department of Physiology and Pathology, Faculdade de Odontologia de Araraquara, Universidade Estadual Paulista (UNESP), Araraquara, Brazil

**Keywords:** brainstem, exercise, obstructive sleep apnea, hypertension, hemorrhage

The brainstem has an essential role in the maintenance of vasomotor tonus, cardiac output, and consequently arterial blood pressure at physiological levels. Several interconnected neuronal groups provide tonic excitation to the sympathetic nerves, which innervate blood vessels and the heart (Dampney, 1994; Guyenet, 2006). Among these nuclei involved with the generation of baseline sympathetic activity to the cardiovascular system, a cluster of bulbospinal neurons located in the rostral ventrolateral aspect of the medulla oblongata (RVLM) is considered the core of the sympathetic nervous system (Guertzenstein and Silver, 1974; Guyenet, 2006). The RVLM contains a large collection of neurons that innervate the preganglionic sympathetic neurons (SPGNs) monosynaptically (presympathetic neurons). In addition, these neurons are an important convergence point for sympathetic reflexes elicited by cardiopulmonary receptors and descending inputs from the hypothalamus and higher in the neuraxis (Guyenet, 2006). The presympathetic neurons of the RVLM are presumably glutamatergic (Stornetta et al., 2002), but a large subset of them, defined as C1 neurons, also express the entire complement of catecholamine-biosynthetic enzymes inclusive of the epinephrine-producing phenylethanolamine N-methyl transferase (PNMT) (Hokfelt et al., 1974; Ross et al., 1984). The C1 neurons are not exclusively presympathetic as many C1 cells do not innervate sympathetic preganglionic neurons, but target instead of the hypothalamus, basal forebrain, and periaqueductal gray matter.

Not least, the brainstem region also contains several nuclei with interconnected structures that are necessary to produce cardiorespiratory homeostasis. The components of this complex network include many types of neurons and satellite cells and are in a long column in the lateral brainstem that extends from the caudal medulla along the ventrolateral medulla to the dorsolateral pons, and dorsally to the nucleus of the solitary tract (NTS) (Feldman et al., 2013; Machado et al., 2017). Based on functional and anatomical criteria, the ventral respiratory column (VRC) located in the ventral lateral medulla is divided into at least five functionally distinct structures (For details, see Del Negro et al., 2018). A similar, if not overlapping, distribution of neurons has been identified for the cardiovascular control system (Guyenet, 2014). Within the same region of the ventrolateral medulla, cardiovascular physiologists have divided the region into three rostrocaudal regions which contain a complex neural network responsible for the generation, modulation, and integration of the sympathetic activity (For details, see Guyenet, 2006).

In this Research Topic in *Frontiers in Physiology*, we have 5 original articles and 1 review article based on the topic entitled “*Integrative Physiology: Systemic Hypertension and Respiratory-Sympathetic Coupling*.” The articles drew together an international collection of ideas that highlighted network mechanisms that underlie cardiorespiratory control and their clinical implications. Recent advances in neurobiology have provided novel insights into the cellular and network mechanisms that underlie cardiorespiratory control. The use of slices, *in situ* and *in vivo* preparations, combined with modern genetic, molecular and optogenetic techniques have led to a new level of understanding of the mechanisms that govern not only the normal physiological integration of cardiovascular and respiratory functions but also the mechanisms that lead to pathologies such as those seen in hypertension, sleep apnea associated with obesity, inflammation, and fetal undernutrition.

The first article “*Medullary Noradrenergic Neurons Mediate Hemodynamic Responses to Osmotic and Volume Challenges*” by Marques et al. provides experimental evidence based on loss of function pharmacological technique that both the A1 and A2 catecholaminergic neurons of the brainstem are essential to hypertonic saline-induced cardiovascular recovery in hypovolemia. Briefly, simultaneous A1 and A2 dysfunctions could impair the efficacy of hypertonic saline-induced recovery in a condition of hemorrhage. The authors discussed based on the literature and previous work from their laboratories that both A1 and A2 cells project to forebrain regions involved in hydroelectrolytic adjustments, and lesion of these groups could lead to signaling deficit and impairment of the recruitment settings of other pathways before the attenuation of hypertonic saline-induced recovery after hemorrhage.

The second article authored by Kim et al. entitled “*Leptin Receptor Blockade Attenuates Hypertension but Does Not Affect Ventilatory Response to Hypoxia in a Model of Polygenic Obesity*” provides key information about the link that obesity can cause hypertension and exacerbates sleep-disordered breathing (SDB). The authors used New Zealand obese mice, a model of polygenic obesity, that have high levels of circulating leptin and hypertension, and are prone to develop SDB, similarly to human obesity. The data showed that leptin receptors blockade attenuated hypertension without exacerbating obesity or SDB, suggesting that obesity-related hypertension is, at least in part, related to leptin signaling. The article highlights the potential applicability of pharmacological blockade of leptin signaling as a therapy for patients with obesity-induced hypertension.

Obstructive sleep apnea (OSA) is a common breathing disorder affecting a significant percentage of the adult population. OSA is an independent risk factor for cardiovascular disease; however, the underlying mechanisms are not completely understood. The data presented by Cetin-Atalay et al. investigated the effect of intermittent hypoxia on endothelial cell activation, characterized by the expression of inflammatory genes, which plays an important role in the pathogenesis of the cardiovascular disease. They demonstrated that pharmacological inhibition or adrenalectomy or carotid body ablation was able to prevent hypoxia-induced endothelial cell activation. The data also elucidated that catecholamines alone were sufficient to cause endothelial cell activation Together, the results suggested that IH does not directly induce EC activation but does so indirectly via the release of catecholamines. Targeting intermittent hypoxia-induced sympathetic nerve activity and catecholamine release may be a potential therapeutic target to attenuate the cardiovascular disease effects of OSA.

The fourth article entitled “*Moderate Physical Exercise Activates ATR2 Receptors, Improving Inflammation and Oxidative Stress in the Duodenum of 2K1C Hypertensive Rats”* by da Silva et al. brings important information that besides cardiovascular and renal impairments, 2 kidney-1clip (2K1C) hypertension model also elicit gastrointestinal dysmotility. In the article, the authors investigated the effect of physical exercise on inflammation, stress biomarkers, and angiotensin II receptors in the duodenum of 2K1C rats. Using standard physiological techniques, it was demonstrated that 2K-1C hypertension elicits an oxidative stress and inflammation process in the duodenum, and physical exercise modulates the expression of the angiotensin 2 receptors (ATR2), suggesting a possible anti-inflammatory and antioxidant effects induced by exercise.

The last original article of the *Frontiers in Physiology* Research Topic of *Integrative Physiology: Systemic Hypertension and Respiratory-Sympathetic Coupling* was authored by Janes et al. and was entitled “*Testosterone supplementation induces age dependent augmentation of the hypoxic ventilatory response in male rats with contributions from the carotid bodies*.” In hypogonadal men, testosterone supplementation may increase the risk of sleep-disordered breathing; however, the site of action is unknown. The present study elegantly demonstrated that (a) androgen receptors are present in the carotid bodies and caudal aspect of the nucleus of the solitary tract; (b) testosterone propionate treatment produced further increase in the respiratory frequency under hypoxia exposure, as well as a further carotid sinus nerve firing rate, and (c) testosterone supplementation reduced apnea frequency during sleep. The authors concluded from their data that potentiation of the carotid body's response to hypoxia by acute testosterone supplementation does not favor the occurrence of apneas but rather appears to stabilize breathing during sleep.

The review by Mariano et al. entitled “*Fetal Undernutrition Programming, Sympathetic Nerve Activity, and Arterial Hypertension Development*” made a significant review from the literature linking the central and peripheral sympathetic system mechanisms on water and salt intake and blood pressure control in the maternal protein-restricted offspring. The article discussed some issues in order to establish the relevance of perinatal disorders in the origin and development of cardiovascular, metabolic, and psychiatric disorders, whose etiology is still unknown, and which clinical manifestation occurs suddenly and unexpectedly in adulthood. This is an important area for future investigation to address the relationship and better comprehend the concurrent systems phenomenon in an integrative view in fetal programming models.

We thank *Frontiers in Physiology* for supporting this Research Topic and the field of Integrative Physiology. The articles presented in this Research Topic will provide readers with an overview of some emerging ideas related to the central neural control of the autonomic and respiratory functions in physiological and pathophysiological conditions.

## Author Contributions

TM, AT, and EC designed research and draft the manuscript. TM make critical revision in the manuscript. All authors approved the final version of the manuscript.

## Funding

This work was supported by the São Paulo Research Foundation (FAPESP; grants: 2015/23376-1 to TM; 2019/01236-4 to AT; and 2015/23467-7 to EC) and the Conselho Nacional de Desenvolvimento Científicoe Tecnológico (CNPq; grant: 408647/2018-3 to AT). CNPq fellowship (302288/2019-8 to AT and 302334/2019-0 to TM). This study was financed in part by the Coordenação de Aperfeiçoamento de Pessoal de Nível Superior—Brasil (CAPES)—Finance Code 001.

## Conflict of Interest

The authors declare that the research was conducted in the absence of any commercial or financial relationships that could be construed as a potential conflict of interest.

## Publisher's Note

All claims expressed in this article are solely those of the authors and do not necessarily represent those of their affiliated organizations, or those of the publisher, the editors and the reviewers. Any product that may be evaluated in this article, or claim that may be made by its manufacturer, is not guaranteed or endorsed by the publisher.
